# Supplementary material for: Localization, inspection, and reasoning (LIRA) module for autonomous workflows in self-driving laboratories
Source: Commun Chem. 2025 Nov 28;8:384. doi: 10.1038/s42004-025-01770-1 (PMC12663431; doi:10.1038/s42004-025-01770-1)
Supplement: Supplementary file 2 — Supplementary Information [file 42004_2025_1770_MOESM2_ESM.pdf]

Localization, Inspection, and Reasoning (LIRA) Module for Autonomous Workflows in Self-Driving Laboratories

Zhengxue Zhou[1,2], Satheeshkumar Veeramani[1], Francisco Munguia-Galeano[1], Hatem Fakhruldeen[1,2], and and Andrew I. Cooper [1,2*]

[1]Department of Chemistry and Materials Innovation Factory, University of Liverpool, United Kingdom
[2]The Leverhulme Research Centre for Functional Materials Design, University of Liverpool, United Kingdom
*corresponding author: aicooper@liverpool.ac.uk

Supplementary Information

S.1 Supplementary Video 1

The Supplementary Video 1 is available in the link[2].

S.2 Supplementary Video 2

The Supplementary Video 2 is available in the link[3]. The key steps where LIRA performs vision-based localization, inspection, and reasoning throughout the workflow are summarized in Table S1, with corresponding timestamps indicating when each function is executed in the video.

S.3 Supplementary Video 3

The Supplementary Video 3 is available in the link[4]. Figure S1 illustrates the error recovery process demonstrated in Supplementary Video 3, where the robot detects and corrects a translational offset in object placement. At step ①, the robot captures an image from the designated inspection pose. The image is then sent to LIRA, which returns an inspection and reasoning result (step ②). This result is processed by the InspectionHandler function, which determines the appropriate corrective action.

Based on the reasoning result, the robot identifies the offset direction. For example, in Figure S1, the PXRD rack is detected as misaligned toward the back direction. The robot then moves to a predefined position, aligning itself for corrective action (step ③). If the error is classified as recoverable, the robot executes a controlled push to reposition the rack correctly (step ④).

S.4 Fine-Tuning Dataset and Prompt Dictionary

A total of 473 images were used to fine-tune the VLM and an additional 94 unseen images were reserved for testing the fine-tuned VLM. Some images of door manipulation failures were reused, as opening and closing failures exhibit the same characteristics.

To ensure comprehensive coverage of manipulation outcomes, the full dataset includes a balanced distribution of success and failure cases across various object types and failure modes. Specifically, it contains:

Black rack: 85 correct placements, 60 unrecoverable failures, and 82 recoverable misalignments across four directions(left: 20, right: 21, forward: 21, back: 20).

White rack: 85 correct placements, 65 unrecoverable failures, and 79 recoverable misalignments distributed equally across four directions (left: 20, right: 20, forward: 20, back: 19).

PXRD door manipulation: 33 successful openings, 28 failed openings; 22 successful closings, 28 failed closings.

This distribution demonstrates that the dataset encompasses both recoverable (e.g., slight translational offsets) and unrecoverable (e.g., severe rotation or occlusion) placement errors, enabling the fine-tuned model to generalize across a diverse range of real-world manipulation scenarios. The dataset is available in `dataset` folder through the link.

Table S2 presents examples of failure cases from the image dataset and prompts used to fine-tune the VLM for inspection and reasoning tasks. For PXRD Plate and 8-hole Rack, the dataset includes reasoning results of recoverable placement errors, where the robot attempts to correct the misalignment based on reasoning feedback. These errors involve translational offsets that can be adjusted through predefined robotic actions. In contrast, for other targets such as the LCMS rack, NMR

[2]https://youtu.be/jw2xnO2dVXk
[3]https://youtu.be/yKcioGeq1JY
[4]https://youtu.be/fSI5bgTbLyY

Figure S1. Illustration of the error recovery process demonstrated in the Supplementary Video 3, where the robot reasons about a translational offset and executes corrective manipulation.

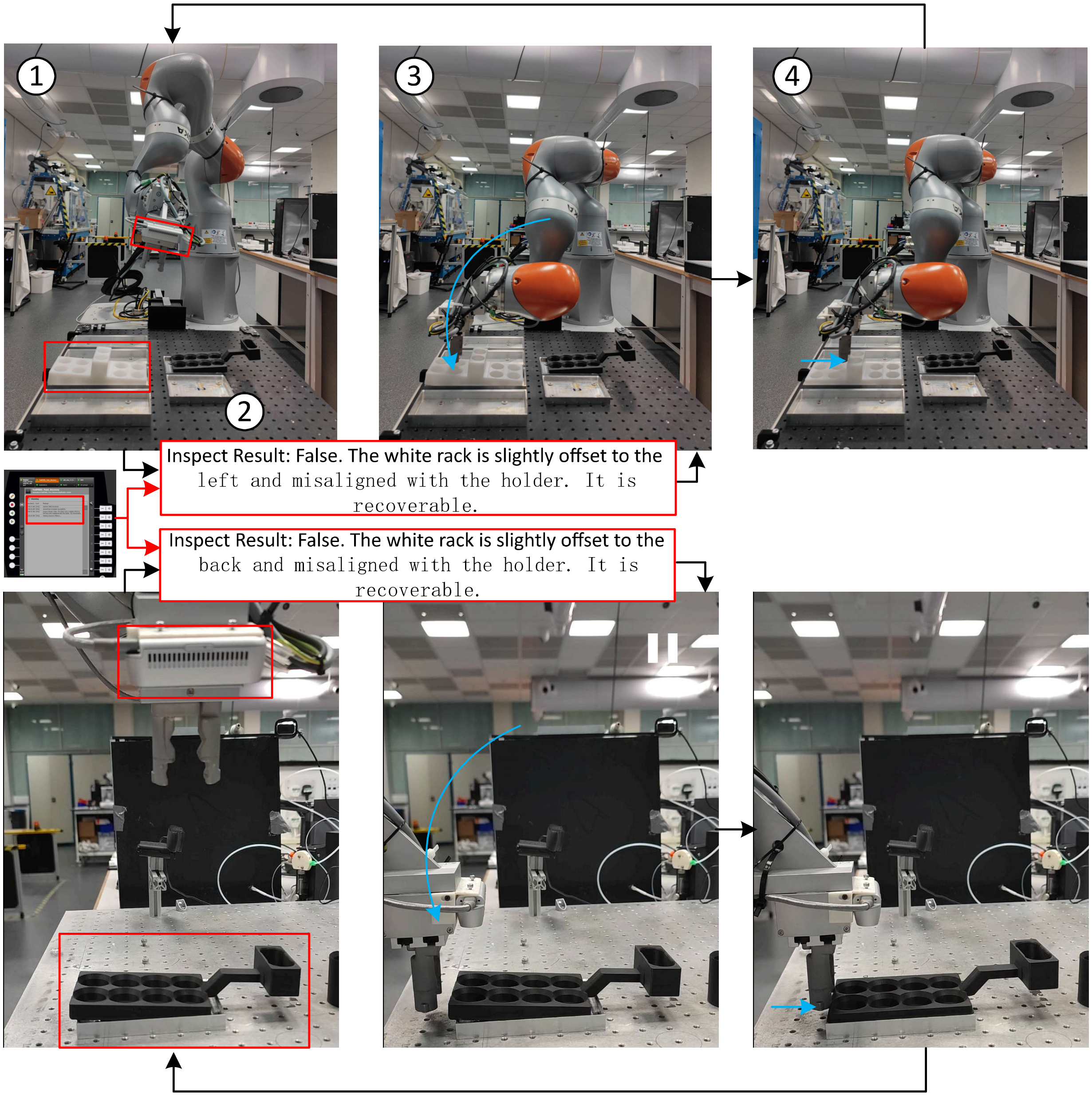

Time	LIRA vision-based functions
0 min 53 sec	Localization at the ChemSpeed station to unload the 8-hole white rack to the robot base.
2 min 36 sec	Reasoning the placement of 8-hole white rack on the robot base.
7 min 14 sec	Localization at the preparation station to load the 8-hole white rack.
8 min 00 sec	Reasoning the placement of 8-hole white rack on the preparation station.
8 min 33 sec	Localization at the preparation station to load the PXRD plate.
9 min 20 sec	Reasoning the placement of the PXRD plate on the preparation station.
9 min 48 sec	Localization at the preparation station to unload the 8-hole white rack to the robot base.
10 min 26 sec	Reasoning the placement of the 8-hole white rack on the robot base.
10 min 46 sec	Localization at the preparation station to unload the PXRD plate to the robot base.
11 min 26 sec	Reasoning the placement of the PXRD plate on the robot base.
14 min 18 sec	Localization at the PXRD station to open the doors.
15 min 54 sec	Reasoning the opening states of doors.
16 min 48 sec	Localization inside the PXRD station to load the PXRD plate.
18 min 26 sec	Reasoning the placement of the plate inside the PXRD station.
19 min 21 sec	Localization at the PXRD station to close the doors.
20 min 49 sec	Reasoning the opening states of doors.
21 min 22 sec	Localization at the PXRD station to open the doors.
22 min 59 sec	Reasoning the opening states of doors.
23 min 47 sec	Localization inside the PXRD station to unload the PXRD plate.
25 min 44 sec	Reasoning the placement of the PXRD plate on the robot base.
26 min 27 sec	Localization at the PXRD station to close the doors.
27 min 59 sec	Reasoning the closing states of doors.

Table S1. Timeline of LIRA vision-based functions during the solid-state workflow testing.

rack, and PXRD door, once a failure is detected, the system pauses the workflow and wait for human intervention, as these misalignments—especially rotational errors and complex occlusions—are not recoverable by the robot. The table categorizes failure types based on inspection targets and their respective locations, with structured prompts used during model inference. This structured dataset enables the VLM to accurately detect placement failures and determine appropriate responses for automated correction or operator intervention.

S.5 Testing of fine-tuned VLM

To evaluate the performance of the fine-tuned VLM before applying it to the robot, we conducted inference testing on 66 unseen images. The evaluation code is available in the link[1].

The evaluation was performed as follows:

1. Model Setup: The fine-tuned VLM was loaded onto an NVIDIA GPU for inference.

2. Dataset Preparation: A separate test dataset containing 94 images was used, with each sample including:

- An input image of a placement scenario.
- A structured inspection prompt.
- A ground truth response.

3. Inference and Comparison: - The model processed the input images and prompts, generating textual responses. - The generated response was compared against the ground truth using a string similarity metric. - Responses were marked correct if their similarity score exceeded a threshold of 0.9.

4. Performance Metrics: - The model achieved an overall success rate of 97.87%, correctly classifying all 94 samples.

Users can re-run the inference testing by following the provided instructions and dataset structure in the link[1]. Our testing result for the fine-tuned VLM is shown in `test_results.json`. The testing result for the pretrained VLM is shown in `test_results_zeroshot.json`

Figure S2. Flowchart of the recovering procedure.

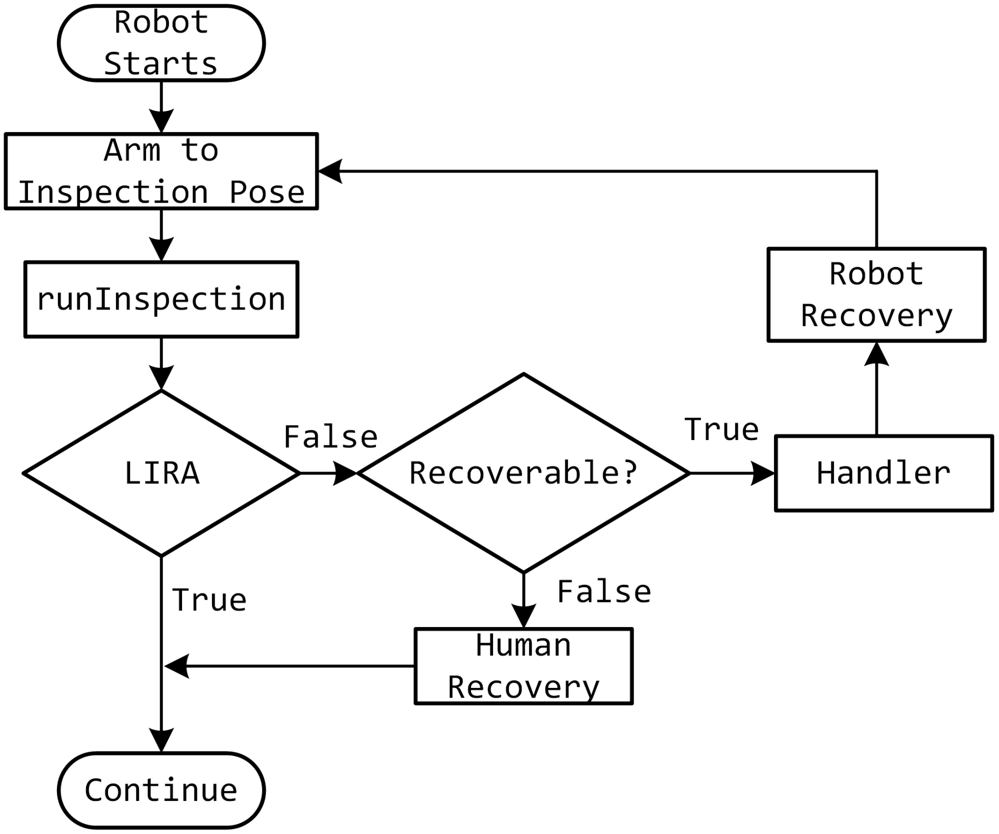

S.6 Training of YOLOv11

To establish a baseline for comparison with our VLM, we trained a YOLOv11 classification model using the same training and test sets. The dataset was prepared in a directory-based format, with images organized into subfolders corresponding to their ground truth classes. A 20% validation split was reserved during training to monitor generalization.

The YOLOv11 model was initialized from the pre-trained `yolo11n-cls.pt` checkpoint and fine-tuned for 300 epochs using a batch size of 32 and an input resolution of 384×384 pixels, matching the resolution used by the VLM encoder (`google/siglip-so400m-patch14-384`). Training was conducted on the same GPU as the VLM.

Following training, the model was evaluated on the same held-out test set used for the VLM: images spanning 18 visual inspection classes. The YOLOv11 model achieved a top-1 classification accuracy of 86.32%, indicating solid performance in a traditional CNN-based pipeline.

However, as discussed in the main text, the YOLOv11 model underperformed in fine-grained inspection tasks, especially those involving subtle, recoverable placement errors. One contributing factor is the limited size of the training dataset—only 473 labeled images were available for 16 distinct classes. This data scarcity poses a significant challenge for CNNs, which typically require large amounts of labeled data to generalize well. In contrast, the VLM benefits from strong pretraining and zero-shot generalization capabilities, allowing it to better capture spatial subtleties and semantic relationships even with limited task-specific supervision. The complete list of 16 visual inspection classes used in the training and evaluation is shown in Table S3. Each class label follows the format `[object]_[status]_[attribute]`, where `black` or `white` denotes the rack type, `true` indicates correct placement, and `false_rec_[direction]` specifies a recoverable misalignment in a particular direction (`left`, `right`, `back`, or `forward`). Similarly, `false_unrec` denotes unrecoverable errors. Door states are labeled as `door_open_true/false` and `door_close_true/false`, reflecting proper or improper open/close operations. The model weight and inference code are available in the link[1].

S.7 Inspection Handler

The Inspection Handler is a critical component of the LIRA framework, responsible for processing vision-based inspection and reasoning results from the VLM. This module determines whether the robotic manipulator should proceed with its workflow,

initiate an error recovery action, or pause for human intervention.

Both the Supplementary Video 3[4] and Figure S2 illustrate the error recovery process executed by the robot using the Inspection Handler. When the robot performs an inspection, it sends a natural language prompt to LIRA, which returns an evaluation result. If the response is `"True"`, the robot continues its workflow. If the response is `"False"`, the system analyzes the reasoning output to determine whether the error is recoverable.

For recoverable errors, such as translational placement offsets, the handler extracts directional information (e.g., left, right, forward, or backward) and triggers predefined correction movements using the robotic arm. The correction process involves moving to a predefined pose and applying a controlled push to realign the misplaced object. After that, the robot will repeat the inspection.

For non-recoverable errors, such as excessive misalignment or rotational offsets, the handler pauses the workflow and awaits human intervention. The system logs the error and notifies the operator for manual correction.

The Java implementation (InspectionHandler.java[1]) demonstrates how these components interact to enable closed-loop error recovery.

S.8 Reproduce

To reproduce LIRA, please follow the steps of the repository[1].

Inspect Targets	Location	Image	Prompt
PXRD Plate	Preparation Station	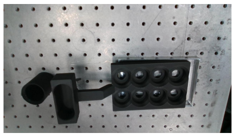	Q: "Has the black rack been placed onto the holder properly?"<br>A: "False. The black rack is slightly offset to the right and misaligned with the holder. It is recoverable."
PXRD Plate	Robot base	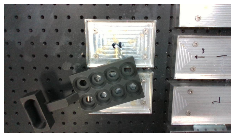	Q: "Has the black rack been placed onto the holder properly?"<br>A: "False. The black rack is slightly rotated and misaligned with the holder. It's unrecoverable."
PXRD Plate	PXRD	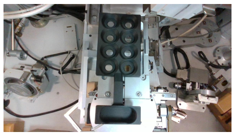	Q: "Has the black rack been placed onto the holder properly?"<br>A: "False. The black rack is slightly offset to the left and misaligned with the holder. It is recoverable."
8-hole Rack	Preparation Station	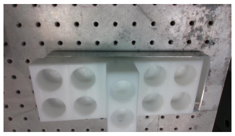	Q: "Has the 8-hole white rack been placed onto the holder properly?"<br>A: "False. The white rack is slightly offset to the left and misaligned with the holder. It is recoverable."
8-hole Rack	Robot base	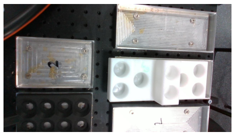	Q: "Has the 8-hole white rack been placed onto the holder properly?"<br>A: "False. The white rack is slightly offset to the left and misaligned with the holder. It is recoverable."
8-hole Rack	Chemspeed	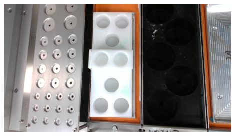	Q: "Has the 8-hole white rack been placed onto the holder properly?"<br>A: "False. The white rack is slightly offset to the left and misaligned with the holder. It is recoverable."
Door	PXRD	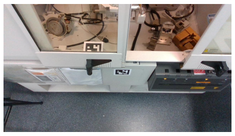	Q: "Has the door been closed properly?"<br>A: "False."
NMR Rack	NMR	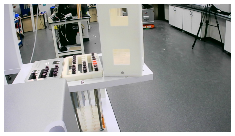	Q: "Has the white long rack been placed onto the holder properly?"<br>A: "False. The white long rack is slightly tilted and misaligned with the holder. It's unrecoverable."
LCMS Rack	LCMS	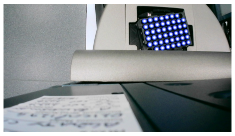	Q: "Has the black rack been placed onto the holder properly?"<br>A: "False. The black rack is slightly rotated and misaligned with the holder. It's unrecoverable."

Table S2. Examples of failure cases and the reasoning outcomes of LIRA.

Class Name	Class Name
black_true	white_true
black_false_unrec	white_false_unrec
black_false_rec_left	white_false_rec_left
black_false_rec_right	white_false_rec_right
black_false_rec_back	white_false_rec_back
black_false_rec_forward	white_false_rec_forward
door_open_true	door_open_false
door_close_true	door_close_false

Table S3. List of all visual inspection classes used for training and evaluation.
