# Supplementary material for: Localization, inspection, and reasoning (LIRA) module for autonomous workflows in self-driving laboratories
Source: Commun Chem. 2025 Nov 28;8:384. doi: 10.1038/s42004-025-01770-1 (PMC12663431; doi:10.1038/s42004-025-01770-1)
Supplement: Supplementary file 3 — Description of Additional Supplementary Files 9requested by the editor) [file 42004_2025_1770_MOESM3_ESM.pdf]

Description of Additional Supplementary Files

File name: Supplementary Video 1

Description: The video showcases the operational stability evaluation of the LIRA module during a prolonged vial transfer workflow. The robot repeatedly localizes itself using small ArUco markers, manipulates racks and vials across multiple stations, and adapts to navigation-induced errors from the mobile base. The process highlights key stages including rack loading, vial transfer, PXRD plate handling, and door operations, each requiring vision-based localization and reasoning to ensure precise execution. Over the course of continuous operation, the system demonstrates its robustness, successfully completing complex sequences of pick-and-place manipulations and station interactions without failure, thereby confirming its reliability for long-term autonomous experiments.

File name: Supplementary Video 2

Description: The video demonstrates LIRA's vision-based workflow for a solid-state experiment, where the robot repeatedly performs cycles of localization and reasoning at different stations. It begins at the ChemSpeed station, unloading and placing an 8-hole white rack onto the robot base, then proceeds to the preparation station for loading and placing both the rack and a PXRD plate. After multiple handovers between the robot base and preparation station, the workflow advances to the PXRD station, where the robot localizes and reasons about door operations, loads the PXRD plate inside, and later unloads it back to the robot base. The sequence concludes with final door-closing actions at the PXRD station. Across these stages, LIRA consistently localizes objects or station states and reasons about their correct placement or status, ensuring reliable execution of rack transfers, plate handling, and station operations in a realistic laboratory setting.

File name: Supplementary Video 3

Description: The Error Recovery Video illustrates the error recovery process executed by the robot using the Inspection Handler. When the robot performs an inspection, it sends a natural language prompt to LIRA, which returns an evaluation result. If the response is True, the robot continues its workflow. If the response is False, the system analyzes the reasoning output to determine whether the error is recoverable.
